# Supplementary figures and images for: Longitudinal observations of the effects of ischemic stroke on binaural perception
Source: Front Neurosci. 2024 Feb 28;18:1322762. doi: 10.3389/fnins.2024.1322762 (PMC10936579; doi:10.3389/fnins.2024.1322762)

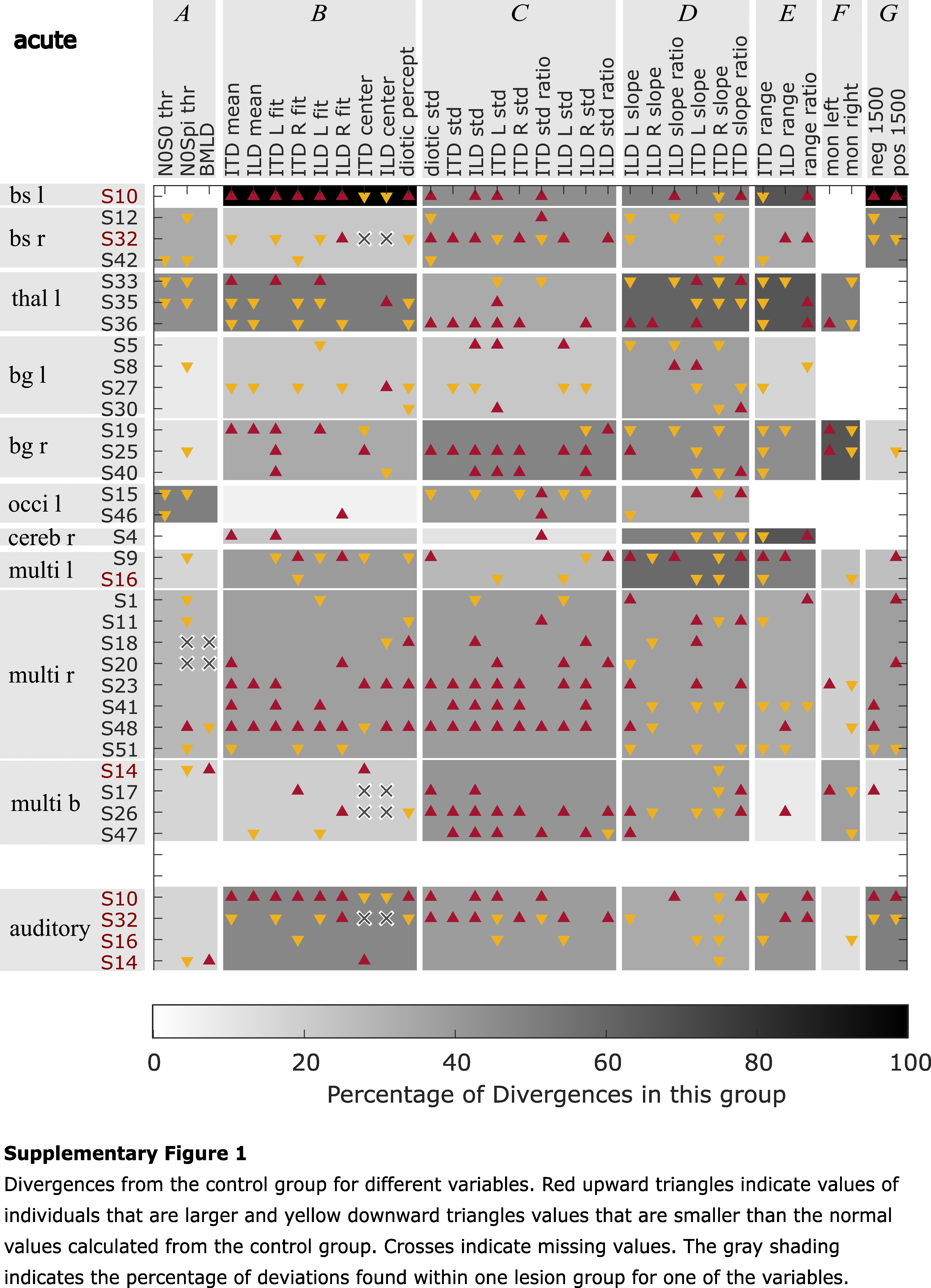

Supplement: Supplementary file 1 [file Image_1.jpg]

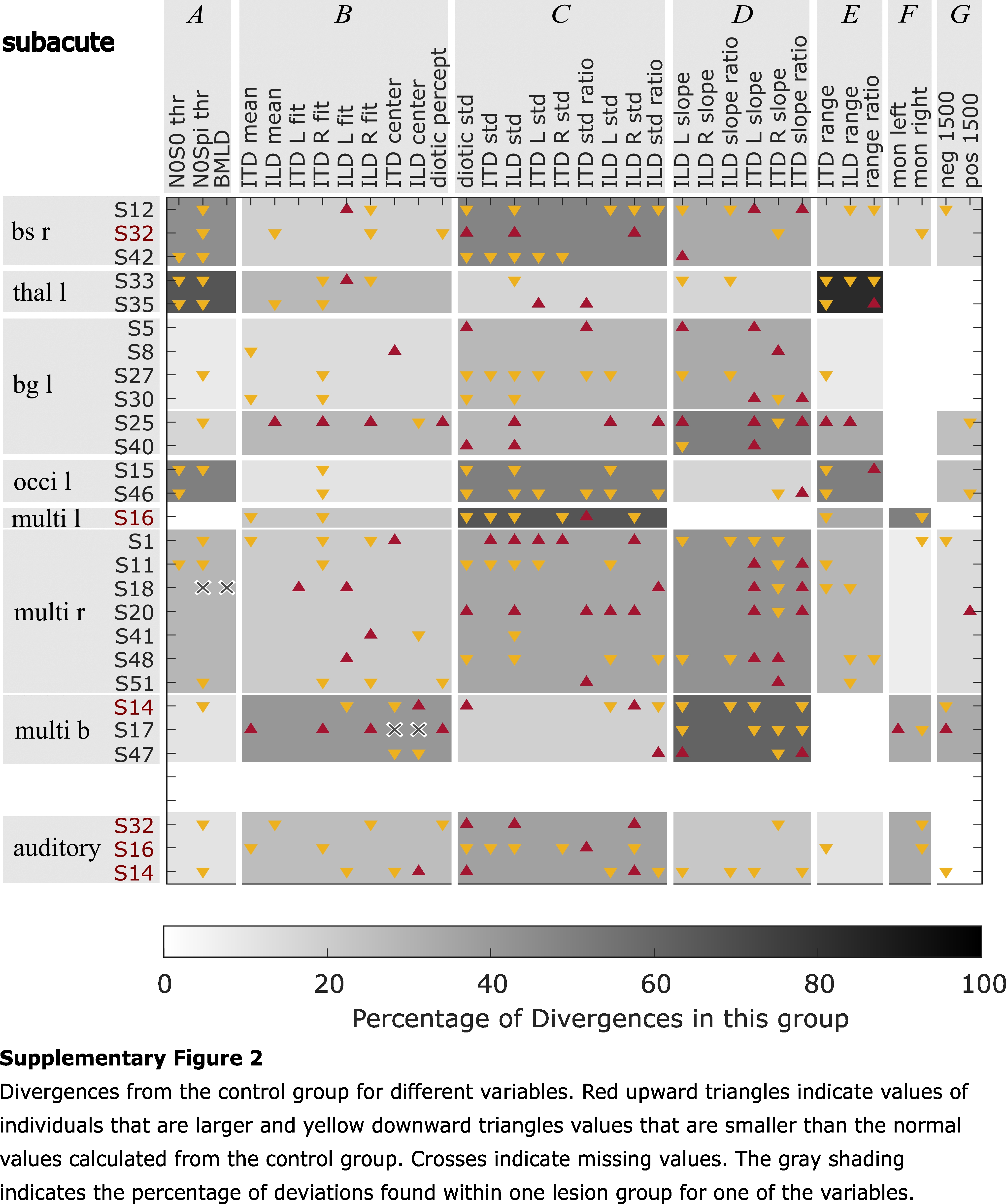

Supplement: Supplementary file 2 [file Image_2.jpg]

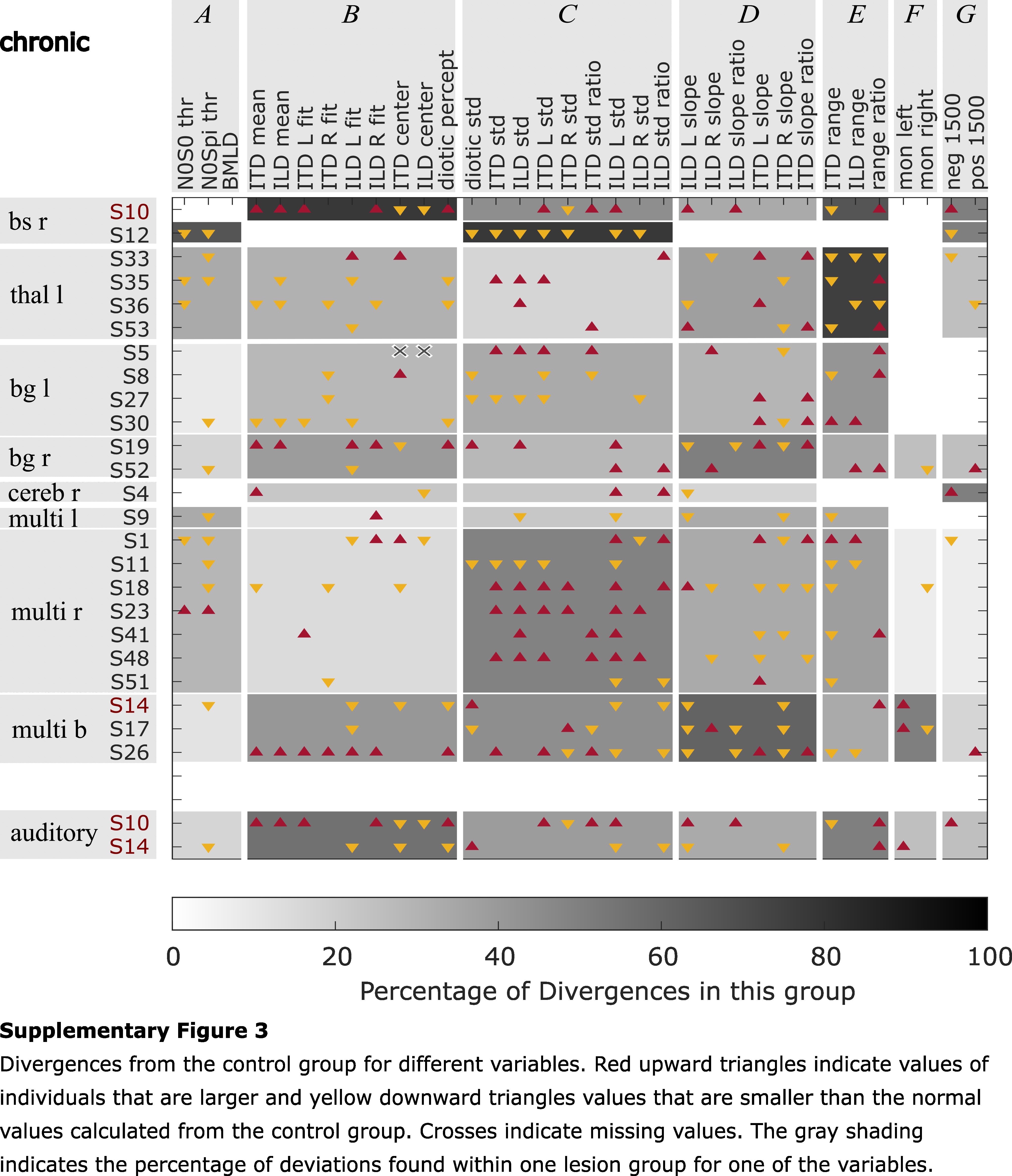

Supplement: Supplementary file 3 [file Image_3.jpg]
